# Towards an inclusive Open Science: examining EDI and public participation in policy documents across Europe and the Americas

**DOI:** 10.1098/rsos.240857

**Published:** 2025-04-30

**Authors:** Natascha Chtena, Juan Pablo Alperin, Esteban Morales, Alice Fleerackers, Isabelle Dorsch, Stephen Pinfield, Marc-André Simard

**Affiliations:** ^1^ School of Publishing, Simon Fraser University, Vancouver, British Columbia, Canada; ^2^ Research Centre for Media and Journalism Studies, University of Groningen, Groningen, The Netherlands; ^3^ Department of Media Studies, University of Amsterdam, Amsterdam, The Netherlands; ^4^ ZBW - Leibniz Information Centre for Economics, Kiel, Schleswig-Holstein, Germany; ^5^ Research on Research Institute (RoRI), London, UK; ^6^ The University of Sheffield, Sheffield, UK; ^7^ École de bibliothéconomie et des sciences de l’information, Université de Montréal, Montreal, Quebec, Canada

**Keywords:** Open Science, science policy, EDI, public participation

## Abstract

National, international and organizational Open Science (OS) policies are being formulated to improve and accelerate research through increased transparency, collaboration and better access to scientific knowledge. Yet, there is mounting concern that OS policies do not effectively capture the ethos of OS, and particularly its goal of making science more collaborative, inclusive and socially engaged. This study explores how OS is conceptualized in emerging OS policies and to what extent notions of equity, diversity and inclusion (EDI), as well as public participation are reflected in policy guidelines and recommendations. We use a qualitative document research approach to critically analyse 52 OS policy documents published between January 2020 and December 2022 in Europe and the Americas. Our results show that OS policies overwhelmingly focus on making research outputs publicly accessible, neglecting to advance the two aspects of OS that hold the key to achieving an equitable and inclusive scientific culture—namely, EDI and public participation. While these concepts are often mentioned and even embraced in OS policy documents, concrete guidance on how they can be promoted in practice is overwhelmingly lacking. Rather than advancing the openness of scientific findings first and promoting EDI and public participation efforts second, we argue that incentives and guidelines must be provided and implemented concurrently to advance the OS movement’s stated goal of making science open to all.

## 1. Introduction

The Open Science (OS) movement has gained traction in recent years, with OS policies being enacted at national, international, regional and institutional levels. At its core, OS is rooted in principles of universal access, participation and transparency, enabling others to collaborate in, contribute to, scrutinize and re-use research while spreading knowledge as widely as possible [1,2]. It is conceptualized as an indispensable tool for the democratization of knowledge through the opening of resources, infrastructures, data and publications to a wide range of social agents [3–5]. While definitions and conceptualizations of OS vary across disciplines and stakeholder groups [2,6,7], in its most holistic definition, OS is defined ‘as an inclusive construct that combines various movements and practices aiming to make multi-lingual scientific knowledge openly available, accessible, and reusable for everyone’ [5, p. 7]. Crucially, this understanding adopts a broad interpretation of ‘science’, akin to the German concept of Wissenschaft, which encompasses not only the natural and physical sciences but also the humanities and humanistic social sciences. Although the OS movement is diverse, its proponents share the key assumption that promoting ‘openness’—of multiple things, for multiple groups of people, and at multiple levels and geographies—will increase transparency, enhance trust and encourage innovation, as well as foster equity and widen participation in the scholarly community [8,9].

Governments, funding agencies and research institutions worldwide have begun to support the idea of ‘openness’ as a crucial component of scientific research, often through open access (OA) mandates that require researchers to make their published research available in OA. Countries such as Colombia [10] and Ukraine [11] have also implemented national OS plans, while others, like Kenya and Venezuela, are in the process of drafting their own at the time of writing [12]. In addition, international organizations have issued recommendations and policies for the development and implementation of OS practices. These include multi-lateral organizations like UNESCO [5] and OECD [13], the supranational [14], as well as international scientific societies and professional associations like the International Science Council [15], the World Academy of Sciences [16], and the Association of European Research Libraries [17]. While in Europe the EC has been a driver of OS implementation [18], Latin America has adopted a more grassroots approach through smaller scale initiatives at the national and institutional levels, rather than large organizational efforts [19].

Equity, diversity and inclusion (EDI) are increasingly recognized as fundamental to the success of OS [18,20–23]. From its inception, OS has been envisioned as a pathway to widening participation in science and equalizing the playing field, ensuring that even researchers in resource-poor countries and institutions can access and build on existing research [9,24,25]. In addition to goals such as increased transparency, efficiency and innovation, OS aims to break down barriers to knowledge production and dissemination, fostering a more inclusive and globally accessible research ecosystem [18,26]. This inclusivity is grounded in the principle that openness enables broader participation, inviting other actors—such as community organizations, patient advocacy groups and members of the public—to engage with and contribute to the scientific process [27–29].

While these goals highlight OS’s potential to foster a more equitable and inclusive research landscape, scholars have cautioned that realizing this vision requires careful consideration of structural and systemic inequities. Without adequate guidance and planning, the implementation of OS policies may inadvertently exacerbate inequalities between well-resourced and less-resourced institutions, senior and junior scholars, and well-funded disciplines (e.g. medicine, STEM) and poorly funded ones (e.g. humanities) [18,20,30]. It could also reinforce knowledge hierarchies that place the Global North at the centre of knowledge production and the Global South as the site where this knowledge is consumed [27,31]. Organizations such as UNESCO have sought to address these concerns by including explicit recommendations about ensuring equity, diversity and inclusion in OS [32]. Yet, it is unclear to what degree such EDI-related considerations feature in the broader OS policy landscape.

Furthermore, scholars have cautioned that taken-for-granted assumptions can inform how policy problems are identified, legitimize certain policy solutions while marginalizing others and—in the case of OS specifically—define some research outputs and practices as more valuable than others [8,33,34]. For instance, many policy documents focus on OA and open data (OD), framing them as the central objectives of OS and prioritizing them for investment [31,33]. This focus is probably driven by the alignment of OA and OD with measurable outcomes, such as increased access to publications and datasets, and their compatibility with existing infrastructures and funding mechanisms. By contrast, dimensions of OS that emphasize public engagement and participation, such as science communication efforts that make research knowledge accessible and useful [35,36], and citizen and community science initiatives that invite non-scientists to contribute to conducting research [37–39], are rarely set as policy objectives [12,40].

However, the limitations of prioritizing OA and OD over more participatory approaches are becoming increasingly apparent. Studies on Open Government Data suggest that while such initiatives can enhance transparency and innovation, their outcomes for broader communities remain inconsistent [41,42]. Similarly, while OA has succeeded in increasing the accessibility of research outputs, its transformative effects on society beyond academia have been modest. A scoping review by Cole *et al*. [43] found little evidence linking OA to tangible societal changes. By contrast, participatory approaches such as citizen science (CS) demonstrate significant and diverse societal benefits. These include fostering public trust in science, enhancing scientific literacy and empowering communities to address local challenges [44–46]. These findings suggest that prioritizing access alone is insufficient to achieve the broader goals of OS. The historic lack of emphasis on public participation within OS frameworks may stem from a traditional focus on one-way communication of scientific findings. Arguments for the public benefits of OA and OS have largely framed scientists as producers of knowledge and stakeholders—such as citizen scientists, journalists and clinicians—as passive recipients.

Knowledge co-creation and exchange between scientists and non-scientists has been much less debated in the OA/OS space until fairly recently [27,47,48]. The COVID-19 pandemic underscored the importance of strengthening collaborations between scientists and non-scientific actors [49,50], highlighting the value of involving citizen and community stakeholders in the scientific process [27,51,52]. The UNESCO Recommendation on Open Science [5], the first international standard-setting instrument on OS, highlights the importance of opening science to society in the description of the key pillars of OS—particularly, the pillars of ‘open engagement of societal actors’ and ‘open dialogue with other knowledge systems. This marks a growing recognition of the need to move beyond access-focused initiatives and towards more participatory and inclusive practices.

By focusing on public participation and societal engagement alongside EDI, this study contributes to the growing body of literature that critically examines the evolving landscape of OS policy. While OA and OD remain central pillars of OS, their limitations in achieving meaningful societal impact highlight the need for policies that prioritize engagement and participation alongside access [41,42]. This research aims to provide insights into how OS policies conceptualize and operationalize EDI and public participation, examining the tensions between the stated goals of openness, accessibility and equity in OS, and the practical implementation of these goals.

To this end, the study adopts a critical policy analysis approach informed by Jasanoff’s [53] co-production framework to analyse 52 policy documents from Europe and the Americas published during the COVID-19 emergency, interrogating whose voices and perspectives are included, what omissions and silences persist, and how these gaps shape the framing and priorities of OS policies.

## 2. Defining equity, diversity and inclusion in Open Science

In this study, we use the term ‘EDI’ to refer to the underlying principles of equity, diversity and inclusion in the context of OS, rather than the institutional or organizational frameworks typically associated with EDI (or DEI) initiatives. Drawing on the work of Sandra Harding [54–57], Boaventura de Sousa Santos [58] and Sheila Jasanoff [53,59], we highlight EDI as an ethical foundation for fostering equal participation and representation in science. Harding emphasizes epistemic diversity and marginalized perspectives, Santos advocates for epistemic pluralism and justice to dismantle global power hierarchies in knowledge production and distribution, and Jasanoff underscores the role of public inclusion in democratizing science and aligning it with societal needs. While these thinkers use distinct terms, we adopt the term ‘EDI’ for its broader recognizability and alignment with contemporary discussions about participation, representation and fairness in science.

By situating these diverse conceptualizations under the umbrella of ‘EDI’, we emphasize equity in access to research resources, diversity in epistemological perspectives and inclusion in both scientific knowledge production and public engagement. This approach distinguishes our focus on the ethical dimensions of EDI in OS from institutional frameworks that primarily address organizational policies and practices aimed at improving diversity within institutions.

## 3. Literature review

### 3.1. Previous research on Open Science policy

While a wealth of literature has analysed OA and OD policies at the local, national and international levels, research examining integrated OS policies that address multiple aspects of ‘open’ including open-source software and open education—and their implementation—remains limited. Manco [60] carried out a literature review of works exploring OS policies published since 2007 in English, Spanish, Portuguese and French, identifying fewer than 80 outputs in total. Of those, a significant proportion were theoretical works, small-scale case studies and works that discuss policy issues only tangentially. Empirical studies typically focus on specific aspects of OS, such as legal and ethical considerations, data sharing, and research recognition and rewards, or on policies by a specific type of entity, such as journal policies, institutional policies and national public policies. Additionally, many works analyse individual national contexts (e.g. [61–64]). While studies comparing policies across countries and/or regions do exist (e.g. [19,31,65,66]), they are only marginally represented in the peer-reviewed literature. This lack of comparative studies feeds into a concern that OS policies and their implementation are becoming increasingly universal and context-agnostic [60]. Several scholars have suggested that decisions around when, how and how much to open research can vary widely among institutional, disciplinary and cultural contexts, and that OS policies need to be more sensitive to the diversity of research contexts to which they apply (e.g. [8,67–69]). A universal approach to OS, it is feared, may lead to disparities between researchers and organizations that must follow OS policies without regard to their local capacities and needs.

In terms of research design, only a handful of studies employ qualitative content analysis techniques. Using a discourse analysis approach, Albornoz *et al*. [31] examined the values and assumptions underpinning 49 OS policy documents published between 2012 and 2018 in Canada, Chile, Ghana, Portugal and South Africa. The authors demonstrate how these documents reflect power relations within the scientific community and threaten to reproduce global inequalities in scientific knowledge production and distribution. They also find that policy documents primarily define OS in relation to OA and OD and that ‘using the term Open Science is possibly more so about popularizing the term, rather than pragmatically adapting the system to open practices outside of what is comprised in OA and OD’ [31, p. 4]. Similarly, Manco [19] analysed 31 institutional policies, declarations and statements on OS from research institutions in Brazil, France, Peru and the UK, finding that OS is often used as a proxy for OA and OD and that these components of OS are the ones most developed in the documents examined. Notably, only one out of the 31 documents mentioned EDI as inherent to OS**,** and most framed science communication as a process between researchers, rather than a dialogue with the public. A study of national and organizational OS policy documents from seven European countries identified a similar trend in terms of which OS components are privileged, noting that each country focused on those components of OS that aligned with its capacities and strategic priorities [70].

Collectively, the existing literature suggests that OS policies vary widely across geographies, generally focus on only two forms of openness (OA and OD) and seldom consider the contextual factors that are so important to how openness is perceived and practised. The literature also hints that OS policies pay little attention to the mounting concerns about EDI in OS—and even less to forms of openness that seek to invite wider public participation and engagement in science. Importantly, however, this apparent lack of attention to EDI and public participation may simply be an artefact of the methodologies employed by previous research, as few if any studies have explicitly examined these dimensions.

### 3.2. Equity, diversity and inclusion in Open Science

EDI is an ethical and conceptual framework that promotes the fair treatment and full participation of all people, especially populations that have historically been under-represented or subject to discrimination due to factors such as background, identity, gender, religion, race, ability or location [71]. Equity—not to be confused with equality—refers to the principle of fairness and equality in *outcomes*, not just in resources and opportunities [72]. OS initiatives have long recognized equity as a foundational goal, dating back to the early movements that framed OA as a means to dismantle the historical barriers that have perpetuated inequities in scholarly communication [2,7,18,24,73]. More recently, a stakeholder-driven study by Ali-Khan *et al*. [74] found that increased equity was considered a key success factor for OS, while an analysis of OS initiatives in psychology suggests that OS practices like data sharing and collaborative analyses can further equity by mitigating both the financial burden and time constraints of conducting research for under-resourced researchers [75].

Diversity in science refers to the need for stronger representation of individuals from different backgrounds and perspectives in scientific practices and institutions [76]. At the forefront of discussions on diversity in science are generally two types of diversity: identity diversity, which refers to the representation of various facets of identity, such as race, age and gender among individuals within a given group [77], and cognitive or epistemic diversity, which refers to the recognition and validation of diverse ways of knowing and understanding the world that are historically and culturally situated [78]. This includes non-Western knowledge systems that have historically been marginalized and objectified in academia and science more broadly [26,54,79]. Scholars have long argued that OS projects, if planned intentionally, can broaden the diversity of science-producing actors [27,61,80], while a recent study by Gervais *et al*. [81] illustrated how OS tools and practices like OD and preregistration can help legitimize the work done by women researchers.

Lastly, inclusion refers to the act of creating an environment in which any individual or group feels welcomed, safe, supported, respected and valued to participate, regardless of background and identity [82]. It has been suggested that participatory processes like CS could make scientific endeavours more inclusive and understandable for large audiences [83–85]. However, definitions around EDI and how they are operationalized are often controversial [86,87], which may explain why some OS advocates have been cautious about linking OS explicitly with particular definitions or frameworks in this area [88].

As discussed earlier, OS initiatives are increasingly focused on integrating EDI principles into OS practices, as highlighted in the UNESCO Recommendation on Open Science [5], which underscores the importance of equity, inclusivity and diversity in its core pillars, particularly emphasizing the engagement of societal actors and the integration of diverse knowledge systems, including Indigenous and non-Western knowledges. At the same time, it is important to acknowledge that OS policies are embedded within a broader context of power imbalances and historical inequalities in knowledge production [22]. Academia itself is well-documented as an inequitable space, with entrenched structural issues related to racism, ableism, misogyny, ageism and other forms of discrimination [89–91]. These issues shape who gets to participate in science, whose voices are heard, and whose knowledge is validated. Systemic advantages are often afforded to certain groups—particularly those who are white, male, able-bodied and well-resourced—who benefit from greater access to funding, publishing opportunities and institutional support [92–94]. These imbalances can prevent marginalized groups from fully engaging with and benefitting from OS, despite its stated commitment to inclusivity [5,95,96].

Further, implementing OS policies requires capacities (in terms of knowledge, skills, financial resources, political will, technological readiness and motivation) that vary across regions, institutions and demographics [18,20]. A study by Olejniczak & Wilson [97], for example, found that authors who are male, employed at a prestigious university, more advanced in their careers and funded by federal grants were more likely to publish OA by paying an article processing charge (APC). Such examples highlight that the increasing adoption of OS practices, such as OA and OD, will not automatically lead to a more inclusive scientific landscape without a deliberate focus on EDI. Far from being a panacea for systemic inequities, OS practices must be critically examined within the context of these persistent structural barriers.

The willingness of policymakers to integrate EDI principles into science policies, and of researchers to advocate for such integration, is often limited because both groups may benefit from, and even thrive within, inequitable institutional structures [98]. This makes it unlikely that policymakers and researchers will proactively pursue meaningful integration of EDI into OS policy without external pressures, accountability measures or more systemic changes that go beyond voluntary compliance. As such, the implementation of EDI principles in OS policies requires more than goodwill; it necessitates structural interventions that address the inequities embedded within the academic and research landscapes.

### 3.3. Public engagement and participation in science

Beyond making science more inclusive of a diversity of researchers and knowledge systems, OS is also increasingly conceptualized as a way to make science more inclusive of those outside of science. This can be seen in the development of both academic and science policy discourses in the last decade, in which the principle that the public has a right to access scientific knowledge and to participate in its development has been gaining traction [99–102]. Scientists have aimed to put this principle into practice in a number of ways, including *public engagement* activities such as sharing their research in the media and facilitating dialogue with diverse stakeholders to support mutual learning [103,104]. The public’s right to contribute to science has also been enacted through efforts to increase *public participation*—that is, to give more weight to citizens and civil society actors in defining research needs and implementing research and innovation [105]. As public engagement and participation have similar goals, they are often used interchangeably [103,104]. Broadly speaking, they are believed to lay the groundwork for a science that is, as Sayre *et al*. [106] have argued,

*public* in multiple senses of the word: a science whose practices and data are transparent and accessible as broadly as possible, that serves public needs and interests and is receptive to public participation, that is applicable as one of many inputs to policy, and that is communicated in ways that enable it to contribute to those policies and improved quality of life for the citizens who support it. (p. 50)

Wehn *et al*. [102] build on this idea by emphasizing the critical need for societal engagement to be explicitly integrated into OS policies. Their study synthesizes insights from academic literature and identifies opportunities and challenges for embedding societal engagement within regional and national OS frameworks. Importantly, they argue that societal engagement is often underdeveloped compared with measurable aspects like OA and OD, despite its potential to align OS more closely with the values of inclusivity and public participation. This gap reflects broader concerns that while OS policies aim to democratize science, they may fail to address the structural inequities that shape public participation.

Some of the activities that may be used to foster this kind of ‘public science’ include science cafes (i.e. events that encourage open debate between scientists and the general public) and direct involvement of citizens in research activities—e.g. through practices such as community science, CS, and crowdsourcing [36,104,107]. Scholars, however, have pointed out that there are different types and levels of participation and engagement, some more democratic than others (e.g. [108,109]). For example, Wynne [109] has argued that ‘engaging’ the public in two-way dialogue in order to win their trust is not truly an act of listening or mutual learning; it is a way to maintain science’s authority that only strengthens existing power imbalances between those within and outside of science. By contrast to this *deficit model* approach to public engagement [110], activities that cultivate a sense of belonging in science, facilitate equitable collaborations among diverse stakeholders, and encourage members of the public to bring their experiences, critiques, perspectives and questions into conversations about science are believed to be more inclusive and empowering [111]. These activities can take many forms but are generally described as following either a *dialogue* or *participation* model of engagement [110,112].

In other words, *how* public participation and engagement activities are implemented shape the nature, impact and implications of those activities and their potential to advance a more open, inclusive scientific system. In the policy landscape, policymakers often opt for citizens’ participation when they need resources that would otherwise be difficult to obtain [113]. In doing so, they look to participation as a tool which can provide both cognitive and political resources [113], using dialogic or participatory forms of public engagement in pursuit of deficit model goals. Similarly, activities like CS can be an important vehicle for democratizing science and promoting the goal of universal and equitable access to scientific information [114], but they can also perpetuate power differentials when those who have laboured on data collection are not in control of the data [115]. Infusing public engagement and participation activities with OS values to truly make science ‘open’ to all requires intentionally planning for public engagement and participation [40,116]. As these scholars suggest, the successful integration of public engagement and participation into OS requires intentional planning to ensure that diverse publics are meaningfully included in scientific processes and practices.

### 3.4. Research questions

By analysing 52 OS policy documents published in English, Portuguese, Spanish, Greek and German between January 2020 and December 2022, our study aims to answer the following research questions (RQs):

RQ1: How is Open Science defined and conceptualized in OS policy documents?

RQ2: How and in what contexts are EDI mentioned in OS policy documents?

RQ3: How and in what contexts are public engagement and participation mentioned in OS policy documents?

In doing so, the study also examines whether the values of EDI and public engagement/participation are operationalized via concrete and actionable items in these documents to reveal the extent to which they are *actually* prioritized by policymaking actors and whether or not they are treated as essential to advancing OS agendas.

## 4. Theoretical and analytical framework

Our theoretical approach is grounded in critical policy analysis, which examines the power dynamics, normative assumptions and socio-political contexts embedded in policymaking processes [117–119]. Policies, from this perspective, are not neutral tools but social constructs that reflect and influence the values, priorities and discourses of their creators. Unlike traditional analyses, which treat policies as objective instruments for achieving predefined goals, critical policy analysis interrogates how they construct and sustain hierarchies of power, determining what and who is valued, and whose perspectives are marginalized [118,120].

Critical policy analysis often encompasses four primary types of inquiries [119] These include examining discrepancies between what policies claim to achieve and their actual implementation, exploring the historical context and evolution of specific policies, analysing how policies influence the allocation of power, resources and knowledge, and evaluating the role policies play in reinforcing or mitigating broader social inequalities and stratification. Our study aligns most closely with the third and fourth types of inquiry, as it focuses on how OS policies shape the dynamics of participation and representation in scientific knowledge production. Specifically, we examine how core concepts like ‘openness’ and ‘access’ are constructed, whose interests these constructions ultimately serve, and the extent to which OS policies democratize or perpetuate inequities in knowledge production and dissemination.

Within this framework, we employ critical document analysis to interrogate policy documents as both expressions and instruments of power [121]. Critical document analysis draws attention to silences and absences in said documents—issues, voices or perspectives that are omitted, marginalized, or deliberately excluded. By critically examining these silences, we can analyse how concepts such as EDI and public participation are framed—or sidelined.

To deepen this analysis, we draw on Jasanoff’s co-production framework [53], which emphasizes the reciprocal shaping of scientific knowledge and social order. Co-production challenges the traditional separation of scientific and political authority by arguing that how we understand the world and govern it are deeply interconnected [122]. Applied to OS policies, this framework highlights how they embed values like transparency, accessibility and democratization of knowledge while reflecting the cultural and institutional contexts in which they are created. It also examines whether OS policies genuinely foster inclusivity and participation or perpetuate knowledge asymmetries. Further, this approach sheds light on how OS policies negotiate tensions between global norms—such as those articulated by UNESCO [5]—and local contexts, revealing how universal ideals are adapted and mediated in specific regional settings.

In this study, we apply co-production to examine how OS policies function as governance mechanisms that formalize particular visions of openness, inclusion and participation. Rather than treating OS policies as neutral instruments, we analyse how they construct and stabilize specific norms and expectations around openness, shaping whose knowledge is legitimized and how participation is defined. This approach allows us to interrogate the ways OS policies both reflect and actively shape governance structures, embedding particular epistemic and institutional hierarchies into global OS frameworks. By integrating critical policy analysis with Jasanoff’s co-production framework, we provide a theoretical lens to investigate how OS policies might shape, and be shaped by, systemic barriers, power asymmetries and normative assumptions, while remaining attuned to their potential for fostering a more equitable and inclusive vision of open science.

## 5. Study design and methods

This study employs a critical document analysis approach to examine 52 OS policy documents released between 2020 and 2022 across Europe and the Americas. Within this framework, thematic analysis, as outlined by Braun and Clarke [123,124], was used as the analytical method to identify and interpret recurring themes and patterns in the texts. Rather than seeking fixed or objective meanings, this kind of qualitative analysis acknowledges the subjectivity of both the creator and the reader, allowing for multiple, nuanced interpretations. Our approach aligns with previous analyses of OS policy documents [19,31] and broader policy studies (e.g. [125–127]). A detailed overview of our methodology, including sampling, search strategies and analysis procedures, is provided in the following sections.

### 5.1. Sample

Sampling in qualitative document research prioritizes capturing diversity and richness over comprehensiveness [128]. The sample in our study comprises 52 OS policy documents published between 1 January 2020 and 31 December 2022. Sampling concluded in March 2023. These documents reflect Europe and the Americas, regions chosen for the research team’s expertise and familiarity with their socio-political and linguistic contexts. The sample includes documents in English, German, Greek, Portuguese and Spanish—languages spoken by team members conducting the analysis. While this linguistic filter ensured accurate interpretation of the documents, it inherently limited the inclusion of documents from countries where other languages dominate. Additionally, English-speaking regions outside Europe and the Americas (e.g. Oceania) were excluded due to the team’s lack of expertise in those contexts. The timeframe was selected to reflect the most current state of OS policy and extend prior research in this area.

In line with previous research [31,129], we defined policy documents as written documents that contain guidelines, rules, regulations, laws, principles or directions to put OS values and principles into practice. We included documents that sought to create or implement policy, or shape policymaking processes more broadly, including national plans, funder mandates, internal and external organization policies^1^, and policy recommendations by professional organizations and international agencies. By including documents from diverse geographic and policy levels, we aimed to capture the varied visions and priorities of multiple policy actors while acknowledging that our dataset reflects regional and linguistic limitations.

Given the inherently fragmented nature of the OS policy landscape—characterized by the emergence of policies from diverse sources such as national governments, regional collaborations and international organizations—we prioritized capturing the breadth of documents rather than applying overly restrictive criteria. This inclusive approach allows for a holistic overview of the evolving OS policy landscape, ensuring that the analysis reflects the decentralized and multi-stakeholder nature of the OS movement. By including country-specific, regional and international documents, our sampling approach acknowledges the varied scales and contexts in which OS policies are developed and implemented, providing a more nuanced analysis.

While the vast majority of documents in our sample are concerned explicitly with OS policy, we also included documents on public access to research and scientific data published during the pandemic period, as well as OA/OD policy documents by key stakeholders in the OS space (e.g. funders) in the absence of integrated OS policies published by said stakeholders. This methodological choice was made to capture the diversity of emerging nature of OS policy and with the understanding that the term ‘open science’ has varying uptake across regions and stakeholder groups. We excluded institutional policies by research institutions as they have been examined elsewhere (e.g. [19,130]) and because including them would have made it difficult to achieve sufficiency, given the size of the geographic regions being investigated.

Still, over half of documents obtained were from Europe. Roughly one fifth were from international organizations and governing bodies, and the rest from the Americas (table 1). Government ministries or departments published around 30% of the documents, while multi-lateral organizations, academic associations and/or networks, national advisory bodies or coalitions, scientific organizations and private or public funders each published less than 15% of the sample (table 2). A full list of documents can be found at https://osf.io/dgp3z/ [131].

**Table 1.** Distribution of documents by region.

region	total no. of documents	breakdown by country
Europe	29	Austria (1), Bulgaria (1), Cyprus (1), Finland (1), France (2), Germany (1), Greece (1), Hungary (1), Ireland (1), Malta (1), Montenegro (1), The Netherlands (1), North Macedonia (1), Portugal (1), Slovakia (1), Spain (2), Switzerland (1), Ukraine (1), United Kingdom (2), Regional/ Pan-European (7)
international	10	N/A
North America	9	Canada (2), USA (7)
Latin America	4	Argentina (1), Brazil (1), Chile (1), Colombia (1)

**Table 2.** Distribution of documents by policy actor.

type of policy actor	no. of documents
government ministries or departments	18
multi-lateral organizations	8
academic associations and/or networks	7
national advisory bodies or coalitions	6
scientific organizations	6
private/public funders	6

The outsized number of European stakeholders in our sample is in line with what Albornoz *et al*. [31] found in their own analysis of OS policy documents. Europe’s leading role in OS policy development and implementation—e.g. via initiatives like the Open Science Policy Platform (OSPP), the European Open Science Cloud (EOSC), OpenAIRE and cOAlition S—has also been noted in previous research [78]. In addition to the uneven geographic distribution of the documents in our sample, regional differences can be observed in terms of the stakeholders involved in OS policy planning and implementation. For example, we identified several policy documents published by academic associations and scientific organizations in Europe, but almost no such documents in the Americas, where most documents identified were published by government ministries/departments and national advisory bodies. This may be in part due to our search strategy but is probably also indicative of OS being governed differently across different regions. Our search strategy is described in detail in the next section.

### 5.2. Search strategy

We searched for policy documents between July 2022 and January 2023 using several sources including: Google.com, The Council for National Open Science Coordination, bibliographic databases (Policy Commons, Overton), Zenodo.org, the UN Digital Library, recommendations from subject matter experts and reference lists from relevant literature. We used keywords such as ‘open science’, ‘open research’, ‘policy’ and ‘guidelines’ to identify relevant documents, along with equivalents in Spanish, Portuguese, German and Greek. Because policy documents are often labelled using words such as ‘plan’, ‘guidelines’ or ‘strategy’, we also included such synonyms in our search strategy.

As our initial searches yielded few relevant results and we found pertinent documents to be widely dispersed around the web, we adopted a flexible search strategy. Specifically, we identified and added new documents to our sample using a snowballing approach, which leverages existing networks and references to uncover additional materials that would otherwise be difficult to find [132]. We began by consulting key documents and publications within the OS domain, from which we followed citations and references to discover additional relevant documents (i.e. ‘citation-based snowballing’, [133]). Additionally, we navigated through organizational and institutional websites, following links and leads to locate pertinent policy documents. This iterative process enabled us to refine our sample, ensuring a diverse range of perspectives.

To enhance the breadth of our sample, we conducted an informal consultation with four experts within our research network who possess regional expertise in OS. These experts, representing different regions included in our study, were asked to review our preliminary sample and identify any key documents we might have missed. This process yielded two additional documents for inclusion. While our general search strategy already incorporated focused efforts to capture a diverse range of documents across regions, an additional targeted search was conducted specifically for Latin America after identifying its under-representation in the sample. This supplementary effort did not yield any new documents.

To determine when we had reached information sufficiency [134,135], we assessed whether the data collected provided enough detail and richness to address our research questions comprehensively. The evaluation focused on ensuring that the sample captured a range of perspectives and practices, offering sufficient depth to reflect the nuances of the policy landscape within our regions of interest.

### 5.3. Data extraction and analysis

We adopted a hybrid approach using both inductive and deductive analysis [123,136]. Braun & Clarke’s [123] six-phase framework for thematic analysis guided our approach, encompassing familiarization with the data, coding, theme development, refinement, definition and final narrative production, applied across both deductive and inductive stages of analysis.

In the first step of the analysis, we used NVivo’s case functionality to classify documents according to region, country, type of document, type of policy actor and level of policymaking. This allowed us to analyse the documents with respect to broader contextual attributes. Coding, conducted in English for consistency and accessibility, was performed by the first and third authors. The first round of coding was deductive and was based on research questions or prominent themes in the literature. These themes were identified through an initial review of foundational OS frameworks, including the UNESCO Recommendation on Open Science [5], as well as prior studies on OS policies and conceptual work that traces the history, evolution and ethical underpinnings of the OS movement (e.g. [1,31,137]). A list of initial themes was created by the first author and subsequently refined through group discussion and feedback from the research team to ensure that it accurately captured the complexities of the OS policy landscape and aligned with the goals of the study. A list of study themes is available at https://osf.io/dgp3z/ [131].

During the first coding stage, simple nodes like ‘OS definitions’, ‘proposed activities’, ‘gender disparities’ and ‘participation’ were used to locate relevant sections within the documents and to get a better sense of the data. In subsequent coding rounds, we used an inductive approach to identify patterns and interrelationships in the data by means of thematic codes [123], such that new codes and sub-codes were added, deleted and merged in each round of coding.

Our coding was collaborative and iterative, aimed at fostering shared understanding and conceptual alignment. To ensure consistency in interpreting and applying the coding framework, the two coders began by jointly coding a subset of the documents to identify any differences in interpretation and refine our initial set of codes. We then compared our coding and discussed the rationales behind any differing interpretations to refine our framework to reflect the complexity of the data. Throughout this process, we prioritized conceptual coherence, focusing on whether our interpretations aligned with the broader goals of the study and reflected the social and contextual nuances embedded in the documents. Our final codebook is available at https://osf.io/dgp3z/ [131].

To integrate the data from cases and nodes, we utilized NVivo’s query and matrix coding functions to systematically examine patterns and relationships. In line with Saldaña’s [138] recommendations, we complemented digital tools with analogue techniques, such as manual note-taking and diagramming on paper, to support the development of preliminary themes and facilitate a deeper understanding of emerging patterns in the data. These methods allowed us to iteratively refine our coding structure and explore connections that might not have been immediately apparent through digital tools alone. Preliminary themes were outlined in Word and shared with the research team for detailed feedback on their relevance, clarity and alignment with the data, then adjusted based on team discussions.

### 5.4. Positionality and reflexivity in analysis

Reflexivity was a key element of our approach. We regularly discussed how our own backgrounds and perspectives might shape our interpretations, particularly given the regional and disciplinary diversity of the documents in our sample. Specifically, we considered that our team has diverse disciplinary backgrounds—including information science, education and science communication—and that our collective academic and professional experiences have been shaped by our engagements with issues of EDI in the context of OS and beyond. As advocates for a more equitable and participatory scientific system, we acknowledge that our perspectives are informed by these values, which may have influenced the lens through which we interpret the data. Further, several authors are early career researchers (ECRs), women and/or disabled, and their perspectives are shaped by ongoing efforts to navigate systemic inequities within academia. Two of the authors were born and raised in the Global South and/or have extensive professional experience working there, which provides a critical perspective on regional contexts and informs our analysis of OS policies across different geographies. We are also mindful of the privileges and limitations of conducting research from academic institutions situated in the Global North, which may have impacted our access to and interpretation of OS policies from the Global South. Reflecting on and discussing these aspects of our positionality enabled us to remain attuned to the multiple, sometimes competing perspectives and narratives represented in the policies.

## 6. Results

### 6.1. Conceptualizations and definitions of Open Science

As discussed in §3, OS is often narrowly defined within policy documents, typically as a synonym for OA and/or OD. Our analysis, however, draws a more nuanced picture. While the documents analysed generally place OA and OD over other OS components—such as open peer review, CS and OS education/skills development—many also adopt a broader, more inclusive view on OS. For example, in Europe, the *Lindau Guidelines for Global, Sustainable and Cooperative Open Science in the 21st Century* emphasize the importance of global cooperation, public-facing science communication, inclusion of marginalized scholars and capacity building. *SPARC Europe’s Strategic Plan 2021−2024*, meanwhile, highlights open education—alongside OA and OD—as a core component of OS, and an area of major strategic focus for the organization. Along these lines, Slovakia’s *National Strategy for Open Science 2021−2028* notes that OA ‘represents only one aspect of OS’ (p. 9), listing open peer review, open-source software (OSS), open educational resources (OER) and CS as examples. Similarly, in Argentina, national plans include a focus on investing in the ‘the generation and application of various specific tools—research, support, dissemination, public communication or other—for Citizen Science programs and projects’ (p. 17). In Colombia, the national policy outlines a plan to

implement a strategy of public communication of science directed at the different actors and institutions of the SNCTI [National System of Science, Technology, and Information] and to the citizens in their territories, to promote participation in all the processes of generation and use of scientific and technological knowledge, as well as the dissemination and valuation of its results (p. 57).

The *UNESCO Recommendation on Open Science* [5] also appears to have impacted how OS is framed and discussed in subsequent OS policy documents, particularly in Europe and Latin America. In Europe, the *Irish National Action Plan for Open Research 2022−2030*, Slovakia’s *National Strategy for Open Science 2021−2028* and Science Europe’s *Open Science as Part of a Well-Functioning Research System* adopt UNESCO’s definition and reference it several times throughout. In Latin America, Colombia’s *National Policy for Open Science 2022−2031* and Argentina’s 2022 guidelines for the development of a national OS policy (*Diagnóstico Y Lineamientos Para Una Política de Ciencia Abierta en Argentina*) are written in response to, and in concert with, UNESCO’s Recommendation for Open Science. Overall, nine of 15 documents published after the UNESCO recommendation adopt its definition of OS, the majority of which are national plans and policies. In addition, the Spanish Foundation for Science and Technology (FECYT)—while not referencing the definition directly—mentions that all actions included in its 2022−2024 Strategic Plan are based, among others, on the principles of the UNESCO recommendation.

Some documents, however, take a narrower view, focusing on certain components or aspects of OS (e.g. open infrastructure, reproducibility) to the exclusion of others (e.g. citizen science, public engagement). The Greek *National Plan on Open Science*, for instance, notes that ‘Open Science is the new standard for practices, tools and collaboration for producing and distributing scientific output and research results, with a direct scientific, economic and social impact’ (p. 2), emphasizing the importance of national infrastructures to the implementation and furthering of OS without mentioning aspects like science communication. The plan also frames OS as a way to increase Greece’s national competitiveness—both within the European Union, and more broadly—and to strengthen local opportunities for innovation. This framing is found across documents from mid- and lower-income EU countries.

Lastly, several European national OS plans that adopt a broader definition, such as the one put forth by UNESCO [5], ultimately focus on *actions* that promote OD, OA and open infrastructure. That is, there is a clear disconnect in these documents between the broad definition of OS and what is prioritized in terms of implementation. For instance, Ireland’s national OS plan notes that its vision for open research ‘align[s] with and support[s] UNESCO’s definition of the core values of open research’ (p. 4). Yet the three national priorities it outlines are to achieve ‘100%’ OA for publicly funded research, enable Findable, Accessible, Interoperable, and Reusable (FAIR) data principles and embed recognition and rewards for OS into academic policies and procedures.

### 6.2. Equity, diversity and inclusion in Open Science policies

Much like with definitions of OS, there is a mismatch between statements about the importance of EDI and the proposed actions or paths in most of the documents analysed. These documents often include broad statements about the importance of OS for achieving a more just society, but advance policies and recommendations that address only a narrow subset of topics related to EDI. Specifically, documents focus on combating economic, geographic, institutional and career stage-related disparities, with little mention of other disparities (e.g. relating to language, gender and knowledge systems). Similarly, the documents tend to focus on the potential inequitable impacts of a few key developments: transformative agreements negotiated by research institutions, the APC-funded OA market, and commercial deals and market structures. Many also note that OS, if implemented too rigidly and universally, could perpetuate systemic inequalities by ignoring the needs of researchers in the Global South, smaller institutions and industry. By contrast, other developments with the potential to disadvantage particular groups—such as data-sharing mandates that ignore the needs of less well-resourced scholars, or CS projects that only seek to extract free labour from the public—are seldom mentioned, if at all. In other words, the OS policy documents we analysed overwhelmingly embrace EDI in principle but fail to provide concrete guidance on how those values can be translated into practice.

This disconnect between stated values and suggested practice can be seen in the types of documents that most commonly mention EDI: position statements and guiding documents, rather than actual policies and interventions. EDI does feature prominently in some of these documents, such as the *UNESCO Recommendation on Open Science*, the *RDA COVID-19 Recommendations and Guidelines on Data Sharing* and France’s *A Global Strategy for Open Science*. One document—ALLEA’s statement *Equity in Open Access*—is dedicated exclusively to equity, noting that ‘issues of equity and diversity need to be central to any discussion of how the scholarly communication system should be structured’ (p. 2).

This is not to say that EDI is not mentioned at all in other types of documents. EDI is mentioned in some national plans, but it is generally not emphasized as a strategic priority or a core component of OS—at least in Europe and North America. A notable exception is The Netherland’s *NPOS2030 Ambition Document*, which mentions EDI as one of the five ‘core principles’ of OS and argues that ‘diversity, equity, and inclusiveness are crucial for the success of Open Science’ (p. 5). Similarly, some of the Latin American documents examined also emphasize EDI as an essential aspect of OS, particularly in terms of inclusion of citizens and community stakeholders in OS processes and practices. For instance, the *Colombian National Policy for Open Science 2022−2031* lists equality of opportunities as a core principle, arguing that OS ‘should strive to generate conditions for everyone to access scientific knowledge and other knowledge systems’ (p. 36).

What can be seen more readily across many of the documents is an argument for the need to bridge disparities in access and outcomes caused by the unequal distribution of resources between the Global South and North, and between southern and northern countries of the European Union. The European University Association (EUA)’s *Open Science Agenda 2025*, for example, asserts that institutions and countries must receive the support they need ‘to make more OA progress, irrespective of their current situation’, so that ‘everyone has the necessary resources to transition to OA’ (p. 10). A similar sentiment is expressed in the Open Scholarship Initiative (OSI)’s O*pen Science Roadmap: Recommendations to UNESCO*.

Relatedly, concerns about the marketization of OA are also common, with several documents calling for a move away from APCs, aligning with previous scholarship arguing that this model of OA disproportionately disadvantages researchers from certain disciplines or regions of the world, or those who are unaffiliated with an academic institution [139]. For instance, France’s *A Global Strategy for Open Science* cautions against ‘generalizing this kind of model, which generates serious forms of inequality’ (p. 6) within the global research community. It suggests that mechanisms that redeploy funds in favour of OS publishing without publication costs be explored instead. Similarly, Ireland’s *National Action Plan for Open Research 2022−2030*, *BOIA20*, France’s *A Global Strategy for Open Access,* and Argentina’s *Diagnóstico Y Lineamientos Para Una Política de Ciencia Abierta en Argentina* express strong support for inclusive publication and distribution channels, such as society- and academic-led publishing initiatives, OA repositories and OA journals without APCs. These documents also embrace the concept of bibliodiversity, which refers to

supporting and promoting a diversity of publishing actors, a plurality of communication languages, publication formats or funding methods and a variety of levels of intervention (support for local initiatives created by communities) and points of view in a context of greatly varying constraints and capacities for action [140, p. 7].

Related concepts of linguistic diversity and multi-lingualism^2^ are also mentioned in documents such as the *Second French Plan for Open Science, Open Science 2030 in The Netherlands*, *Diagnóstico Y Lineamientos Para Una Política de Ciencia Abierta en Argentina* and Ireland’s *National Action Plan for Open Research 2022−2030*. For example, the *Second French Plan for Open Science* notes that the French government will ‘[e]ncourage multilingualism and the circulation of scientific knowledge by translating publications by French researchers’ (p. 4). Additionally, the Irish and French documents acknowledge the Helsinki Initiative on Multilingualism in Scholarly Communication [141], which advocates for the promotion of language diversity in research. Notably, especially considering the inclusion of documents from 20 non-English speaking countries, France is the only country that places a strong emphasis on multi-lingualism, both in its national OS plan and global OS strategy. Although, it should be said, its planned actions focus on extending the reach of French-language research, not encouraging French researchers to engage with science in multiple languages. Multi-lingualism, in other words, appears to be framed as a strategy for increasing France’s global influence, and not a commitment to linguistically diverse research more broadly. Health Canada’s *Open Science Action Plan* emphasizes linguistic diversity in the context of the Official Languages Act (OLA), reflecting Canada’s legal commitment to promoting English and French as equal official languages. However, it does not make a case for the importance of integrating multi-lingualism or linguistic diversity into OS practices and processes more broadly.

Lastly, factors like race, disability status and gender are hardly mentioned, and when they are, it is only in passing. The same is true of Indigenous inclusion and Indigenous rights (specifically, data rights), which are only discussed explicitly in three of 52 documents (the *RDA COVID-19 Recommendations and Guidelines on Data Sharing, Final NIH Policy for Data Management and Sharing and Supplemental Information* and the *UNESCO Recommendation on Open Science*) and mentioned in passing in two others (Canada’s *Roadmap for Open Science* and Health Canada’s subsequent *Open Science Action Plan*). These gaps suggest a significant gap in current OS frameworks, which often overlook how intersections of identity and power shape access to and benefits from OS initiatives.

### 6.3. Participation and engagement with science in Open Science policies

Overall, the documents analysed recognize the importance of public engagement with science, but the extent and ways in which they do vary widely. Interestingly, while societal engagement is frequently mentioned among the justifications for OS, the public is not always recognized as a key stakeholder of OS. That is, members of the public are more frequently described as potentially *benefiting from,* rather than *contributing to*, OS—aligning with a deficit model of public engagement. (A notable exception here are the guidelines published by Argentina’s Open Science and Citizen Science Advisory Committee in 2022.) This is also reflected in proposed actions and activities, which tend to emphasize providing access to scientific information rather than promoting meaningful participation in scientific endeavours. Policies emerging from Latin America appear more concerned with citizen engagement and involvement compared with other regions; however, due to the sample size, it is hard to draw conclusions that extend beyond the specific documents we analysed.

Across the documents, it is generally acknowledged that scientists have an ethical and moral responsibility to share knowledge with the public in an accessible manner. For example, the *Lindau Guidelines* suggest that ‘[s]cience has a distinct responsibility to communicate its procedures and results to society’ (p. 5). Furthermore, as discussed above, the *UNESCO Recommendation on Open Science* mentions science communication and open engagement of societal actors as key pillars of OS. In response to UNESCO, *The EUA Open Science Agenda 2025* acknowledges global efforts ‘to open the whole research process and bring it closer to society’, adding that ‘EUA will consider opportunities to help its members engage in activities fostering participatory science and openly involving different societal actors, as recommended by UNESCO’ (p. 15).

To integrate citizens in OS, only a few documents emphasize the need to design and implement effective and inclusive science communication strategies beyond academia. For example, Colombia’s national OS policy includes among its strategic priorities the implementation of a science communication plan that ‘promotes participation in all processes of scientific and technological production, dissemination and use’ (p. 50) for scientists and citizens alike. Within the few documents that mention science communication, most position it as a way of improving public epistemic trust [109], which is often framed as a critical issue in contemporary societies [142,143].

In terms of *forms* of participation and engagement described in the documents, CS is by far the most common. CS is mostly mentioned in national plans and related national-level documents, with some governments (mostly in Latin America and Eastern Europe) emphasizing it more than others. The benefits ascribed to CS include community development, increasing public trust and interest in science, fostering scientific literacy and increasing the social relevance of research. Additionally, in documents stemming from Eastern Europe, CS is framed as contributing to national development and reducing disparities between different regions within Europe. However, some documents suggest that the primary goal of CS activities is to aid researchers in their work, using hierarchical language that places scientists in power over laypeople. For example, Hungary’s *Position Paper on Open Science* defines CS as an area of OS ‘where researchers and research communities take the initiative to involve citizens, local communities and the wider society in certain research processes’ (p. 6), while the Slovakian *2021−2028 National Strategy for Open Science* notes that ‘citizen science projects are carried out under the guidance of researchers’ (p. 32). The Slovak plan is one of few European documents that proposes concrete plans for fostering CS, including creating educational materials, engaging students in CS projects, and building a network of cooperation and support for Slovak CS initiatives. The interlinkage of CS with traditional and Indigenous knowledge systems [69,144,145] is notably absent from the documents analysed. This omission suggests a limited recognition of the potential for CS to bridge diverse epistemologies and foster inclusive approaches that integrate knowledge systems traditionally marginalized in mainstream science.

Much like with the documents’ treatment of EDI, we observed a disconnect between abstract support for participation/engagement and the activities proposed to achieve it. For example, Montenegro’s national OS plan mentions ‘collaboration and participation of society’ as a key tenet of OS and notes that ‘Open Science entails a fundamental paradigmatic change where scientific quality implies much more than the published scientific publications’ (p. 10). However, it distinguishes between ‘primary’ pillars of OS (OA, OD and open infrastructure) and ‘secondary’ ones (open methods, open source, open education and citizen science) and organizes its planned activities and operational goals and performance indicators purely around the primary pillars. Additionally, the plan does not list the public among its key OS stakeholders. Similarly, Canada’s *Open Science Roadmap* mentions public engagement third among its justifications for OS, yet its proposed actions focus almost exclusively on OA, OD and scientist-to-scientist communication, reflecting a top-down approach that prioritizes institutional reforms over grassroots engagement. Meanwhile, Ireland’s *National Action Plan for Open Research 2022−2030* mentions developing ‘commitments to embed, within Irish RPOs [Research Perfoming Organizations], the engagement of citizens, broad publics and the end users of research across the entire research process’ (p. 13). However, the document ultimately highlights the need to re-examine rewards and recognition structures to fuel cultural and behavioural changes towards OS—not to foreground public engagement and participation in pursuit of openness. This prioritization indicates that Ireland views cultural and behavioural shifts among researchers as foundational for achieving broader openness, potentially relegating public participation to a secondary role until these systemic reforms are in place.

Nevertheless, several documents illustrate an ongoing effort to include citizens as active stakeholders in the OS ecosystem. FECYT’s *Strategic Plan 2022−2024* positions citizen engagement as central to advancing scientific culture, emphasizing the need to empower citizens to move from passive recipients to active participants in the scientific process, with a focus on promoting critical thinking, enhancing scientific literacy and engaging traditionally under-represented groups. Similarly, the Dutch National Open Science program (NPOS)’s *Open Science 2030 in The Netherlands* argues that in order to ‘create a sustainable and equitable system of knowledge creation and sharing, societal stakeholders should be included in [the] transition [to OS]’ (p. 14) and encourages the use of public engagement and CS projects. The White House Office of Science and Technology Policy’s *Breakthroughs for All: Delivering Equitable Access to America’s Research* similarly notes that

[a]ll members of the American public should be able to take part in every part of the scientific enterprise—leading, participating in, accessing, and benefitting from taxpayer-funded scientific research.

## 7. Discussion

The OS movement aims to ‘make scientific research from all fields accessible to everyone’ [146, p. 2] in pursuit of a scientific system that is not only more efficient, but also more equitable, transparent and beneficial to both science and society [8]. Yet, our analysis of 52 OS policy documents from three geographic regions suggests that there is a lack of policy response for how to turn this vision of an inclusive and participatory scientific system into reality. That is, our results suggest that existing OS policies—while supportive of a wider, more inclusive approach to openness in theory—often fail to provide stakeholders with the guidance needed to put that approach into practice. This lack of concrete guidance is surprising given the importance given to EDI by funders and research institutions [147–149], increasing calls for public engagement in science [32,49,50], and well-documented concerns about the potential for OS to contribute to inequities in science [1,20,21]. It is also detrimental to scientists’ and institutions’ ability to implement practices and strategies that foster more equitable and inclusive outcomes for all communities OS purports to serve. Without concrete guidance, the burden of interpretation falls on individual stakeholders, risking inconsistent implementation and potentially reinforcing existing inequities, particularly for marginalized groups and underfunded institutions.

Our study is, to our knowledge, the first to examine EDI and public participation in OS policy—two dimensions of OS that are seen by many as essential for democratizing science, but which have received little attention within the policy context until now. It is also one of the few to simultaneously analyse multiple geographies and multiple levels of policy design and implementation, offering insights into the visions, goals and priorities of different actors in the OS policy landscape. While our sampling approach limits our ability to make broad generalizations, it does allow us to see both commonalities and differences across regions. The sourced documents were linguistically and geographically diverse, stemming from 24 countries across North America, Europe and Latin America—three regions with unique histories and approaches to OS. We found that OS policy documents in Europe and North America focus on increasing international and transdisciplinary collaboration and developing more effective data sharing systems in order to promote scientific transparency and integrity, further innovation and enhance national competitiveness. This focus reflects their strategic goals of maximizing research impact, rebuilding public trust in science and maintaining leadership in innovation, while also addressing global challenges like climate change and health crises that require coordinated, cross-border efforts and shared resources.

Notably, eastern and southern European national policies often focus on economic growth as a central component of their OS strategies, seeking to strengthen their scientific and technological capabilities. Integration into broader OS infrastructures, such as EOSC, is often emphasized as a means to enhance regional development, improve research quality and increase participation in global scientific networks. This focus may arise from the region’s need to modernize research sectors and enhance competitiveness, particularly in the wake of post-Soviet transitions and the 2008 financial crisis [150,151]. By contrast, Latin American policies focus more on building national capacities through OS. This focus may be rooted in the region’s historical emphasis on higher education and its longstanding view of public research institutions as vehicles for societal transformation, a perspective that gained prominence in the 1960s [152]. During this period, ‘development-oriented’ universities emerged as key players in national development, serving as state-building institutions tasked with preparing professionals, addressing social problems, and contributing to economic and cultural progress. While these institutions have since evolved, Latin America’s public universities—particularly its flagship institutions such as the National Autonomous University of Mexico (UNAM)—continue to fulfil these state-building roles [152]. As Fischman & Ott [152] note, these universities operate with a unique model that balances mass access with a mission to address societal needs, preserve cultural heritage and promote public accountability. A similar model seems to be sought for OS through policies emphasizing efforts to address participation and equity among citizens.

As such, the findings of our study illustrate how countries across various regions emphasize OS differently, responding to national goals (e.g. France’s efforts to increase its global influence) and contexts (e.g. the prevalence of CS in Argentina). In highlighting these nuances, our research provides evidence that OS policymaking, while often influenced by global trends, remains responsive to and shaped by local contexts to some degree. In doing so, this study highlights the importance of context-specific tensions and gaps within OS policy that warrant further exploration—ideally through case studies and analyses of larger and more representative samples.

With respect to definitions of OS, we observed a stark disconnect between how openness is conceptualized in the documents and what is prioritized in terms of action and implementation. That is, many of the documents advance broad definitions of OS that foregrounded engagement with non-academic actors (often drawing on the definition provided in the UNESCO Recommendations on Open Science), but recommend a narrow set of actions focused predominantly on a small subset of open practices, namely OA, OD and open infrastructure. This may change in the coming years with the help of UNESCO’s Open Science Toolkit, which provides practical information for supporting the implementation of its 2021 landmark recommendation documentation—along with the wider adoption of UNESCO’s ‘equitable and inclusive OS’ vision [32]. Yet, the tendency we observed in the documents to select specific aspects of OS and frame them as urgent priorities, while leaving other aspects unaddressed, suggests that this change may require active reorienting of policies rather than occurring organically.

Similarly, although equity, diversity and inclusion were often described as important goals of OS, the documents primarily addressed concerns about APC-based models of OA and the potential for OS to perpetuate existing inequalities between well-resourced and less-resourced regions, countries, research institutions and disciplines. To be sure, these are important issues that warrant consideration in OS policy, but the outsized attention they received may have come at the expense of broader equity-related concerns. Noteworthy is the lack of emphasis on linguistic diversity in the documents, given that scholars have long argued for the importance of communicating scientific findings in local languages in order to combat knowledge inequities within academia [153] and foster wider societal engagement [154]. Similarly notable is the lack of discussion around inclusion of Indigenous and non-Western knowledge in OS practices and methods despite ongoing efforts—led primarily but not exclusively by UNESCO—to help ‘bring about a fair, decolonial Open Science’ [27, p. 1] that serves all people, rather than the interests of a select few.

Along similar lines, the documents often framed widened *access to* research outputs (e.g. OA journal articles, open datasets) as the main—and often only—public benefit of OS. Moving beyond access to ensure that the public can effectively engage with and utilize such outputs was rarely treated as a priority. Instead, documents largely focused on providing material access (i.e. making articles and data open), overlooking the importance of conceptual access (i.e. understandability) for the public to really benefit from science [155]. Most documents also failed to outline opportunities for the public to productively participate in research design and analyses. Of those that did present concrete recommendations for encouraging public engagement, CS was by far the most common means for doing so. However, even in these cases, the documents often framed scientists, rather than citizens, as the primary beneficiaries of CS.

More broadly, we found that public engagement and participation are predominantly framed as a way to build public trust—and thus maintain science’s cultural power and authority—rather than incorporate citizens’ unique perspectives, experiences, knowledge and expertise into science. This highlights how OS policy documents, despite claims of advancing social justice and inclusivity, often perpetuate longstanding power imbalances between scientists and the public [109,156]. Like traditional science communication efforts, they rely on the long-critiqued deficit model of knowledge transfer [110,157], assuming that the public lacks the knowledge or skills needed to contribute meaningfully to science rather than recognizing its potential to enrich and broaden scientific understanding. This dynamic is reflected in Nelhans & Nolin [158], who show how public participation in the EU’s OS transition was largely shaped by institutional actors, with limited engagement from broader societal groups. While OS policies claim to promote openness, their governance structures often prioritize expert-driven transparency over more transformative models of participation. As their analysis of EU policymaking demonstrates, consultation processes tend to reinforce existing hierarchies, sidelining opportunities for deeper public involvement in shaping OS practices. This lack of prioritization of both EDI and public engagement/participation in OS policy documents arguably limits the democratic and emancipatory potential of OS. Part of the current enthusiasm about OS stems from its promises to reform scientific practice in service of the common good, ensure that scientific findings serve the interests and needs of diverse communities, and enhance scientific impact on policy and society [32]. This necessitates moving beyond a focus on improving access to research outputs and recognizing the public as an important actor in science and innovation. However, the reality we documented provides further evidence that OS policies overwhelmingly focus on making research outputs (e.g. publications and data) publicly accessible [31,33,60], neglecting to advance the two aspects of OS that hold the key to achieving a more fair, participatory and inclusive scientific culture—namely, *equity, diversity and inclusion,* and *public participation and engagement*.

From a practical perspective, our findings highlight the need for policies and guidelines that go beyond merely mentioning principles of equity and inclusion and instead provide concrete guidance towards advancing the OS movement’s stated goal of making science open to all [5,159]. Research by the European Commission on Responsible Research and Innovation (RRI) in Horizon 2020 [160] underscores the detrimental effects of inadequate policy guidance and monitoring. Although RRI aims to integrate societal actors into the research process, an evaluation study found that the Directorate-General of the European Commission struggled to fully achieve this objective due to insufficient policy support and guidance [160]. This shortfall highlights the critical need for clear and actionable policies to promote equitable and inclusive OS policies.

Policies that incorporate participatory approaches can democratize knowledge production, align research with community needs and address systemic inequities in access to and use of scientific knowledge. Furthermore, instead of normalizing OS practices like OA and OD first, and promoting EDI and public participation efforts second, we argue that these incentives and guidelines must be provided and implemented concurrently. By linking EDI principles directly to OS policy development, we can ensure that OS becomes a true vehicle for democratizing science rather than reproducing the status quo.

Ideally, equitable and inclusive OS policies would be developed in partnership with diverse stakeholders—including scholars from varied backgrounds and perspectives, as well as other societal actors. As Wehn *et al*. [102] emphasize, effective engagement in OS policies requires embedding societal participation throughout the research life cycle, from agenda-setting to implementation and evaluation. They advocate for clear guidelines and mechanisms that incentivize collaboration between researchers and societal actors while addressing the barriers that often limit participation, such as lack of resources or institutional support. Furthermore, policies should encourage the co-creation of knowledge to ensure that research outputs are not only accessible but also relevant to societal needs and priorities.

As has been suggested by other scholars [8,68,69], and as is backed up by our findings, such policies will need to be context-specific to accommodate the different priorities and realities found across countries and regions. Until policies that prioritize the inclusion and participation of more diverse actors are developed, the OS movement will not be able to truly deliver on its promise to democratize research knowledge.

## 8. Limitations and future directions

This study provides a critical analysis of OS policies through the lens of EDI and public participation. While we have made efforts to ensure transparency and rigour in our methodology, constraints remain. First, the dataset under-represents Latin American policies, despite the region’s progressive stance on OS. This under-representation stems from two factors: (i) the grassroots-driven nature of many Latin American OS initiatives, which are often not formalized as national policies, and (ii) the timeframe of our dataset, which excludes some relevant policies, such as the Public Policy on Open Science published by Mexico’s National Council of Science and Technology (CONACYT) in 2018 [161]. These factors may skew the findings, particularly regarding the role of Latin America in shaping global OS discourse. However, we address this by contextualizing the unique contributions of Latin American countries in §7, acknowledging their significant influence despite the lack of comprehensive formal policies. Second, our sample included policies in four languages and three regions, we excluded other languages and regions (e.g. Oceana, Asia and Africa), narrowing the geographic representation. Future research could broaden inclusion criteria to better capture these regional efforts, including grassroots-driven initiatives and policies that align with OS principles but do not explicitly use the term ‘Open Science’.

Our decision to include a broad range of countries was intended to capture diverse policy landscapes and demonstrate the global dimensions of OS policy. However, we acknowledge that this inclusivity comes at the expense of some depth in contextual analysis and at the national level in particular—though the team members were well-versed in cultural/socio-political contexts of the regions under study. To address this gap, we recommend that future research adopt a more focused approach, for example by targeting countries where EDI acceptance is culturally and politically supported. This could enable a deeper exploration of how such environments foster innovative policy mechanisms for embedding EDI in OS.

Additionally, while our study highlights how UNESCO’s Recommendation on Open Science [5] has been referenced in several OS policy documents, we do not assess the extent of its tangible impact on national and regional OS policymaking. Future research could explore how and to what degree UNESCO’s recommendations are translated into concrete policy actions, rather than serving as aspirational rhetoric. This could be examined through comparative case studies of countries that have explicitly aligned their OS policies with UNESCO’s framework, tracking changes over time to assess implementation and outcomes.

Finally, this study primarily focused on policy documents themselves, without incorporating interviews or additional qualitative data from policymakers, researchers or stakeholders involved in the development and implementation of OS policies. Future research could build on our findings by employing interviews or case studies to examine the policy development process, how different actors negotiate OS principles, and the challenges of implementing EDI and public engagement in practice.

## Data Availability

The datasets supporting this article are available at [131].

## Footnotes

^1^
Internal and external organization policies refer to guidelines issued by organizations to govern OS practices either within their own operations (internal) or for external stakeholders, such as researchers or institutions (external).

^2^
While the documents use the term multi-lingualism, what they describe could perhaps be more accurately described as linguistic plurality: the phenomenon where multiple languages are valued and shared within a community.
